# Inference of Autism Risk Genes Through Comparative Sociogenomics and Molecular Network Analysis

**DOI:** 10.3390/genes17040368

**Published:** 2026-03-25

**Authors:** Alice Chiodi, Ettore Mosca, Francesca Anna Cupaioli, Alessandra Mezzelani

**Affiliations:** National Research Council-Institute for Biomedical Technologies (CNR-ITB), Via Fratelli Cervi, 93, 20054 Segrate, Italy; alice.chiodi@itb.cnr.it (A.C.); ettore.mosca@itb.cnr.it (E.M.); francesca.cupaioli@itb.cnr.it (F.A.C.)

**Keywords:** comparative genomics, social behavior, autism spectrum disorders, gene network, bioinformatics, biostatistics, gene pathways, gene mapping

## Abstract

Background/Objectives: Comparative sociogenomics combines multiple scientific fields to investigate the genetic basis of social behavior across species. Our aim was to uncover the genetic roots of human sociability with possible implications for autism, a neurodevelopmental disorder characterized by social and communication deficits. Methods: We conducted molecular network analysis on 659 sociability-related genes from different animal species, including humans. Results: We identified a network of 240 genes strongly associated with autism (*p* < 10^−15^), with 194 inferred. These genes were grouped into 23 functional communities related to cell–cell junctions and communication, inflammatory and synaptic signaling, neurotransmitter receptors and semaphorin signaling among the more enriched meta-pathways. Some network genes were clustered in nine chromosomal bands (FDR < 0.25), indicating genes’ functional cooperation, shared evolutionary history, and coordinated regulation, and few genes are physically in linkage with ASD genes (within 0.5 cM) or controlled by human-accelerated regions. Conclusions: The most compelling inferred autism risk genes are *MED12, FZD9*, and *DMD* since they are differentially expressed in autistic brains, physically linked to key autism genes, controlled by human-accelerated regions, or mapped to chromosomal regions enriched in network genes. If validated, they could represent novel biomarkers, advancing the understanding of autism’s genetic makeup.

## 1. Introduction

Autism spectrum disorders (ASD) are a group of heterogeneous neurodevelopmental conditions characterized by deficits in social interaction and communication, restricted interests, and repetitive behaviors (DSM-5 (https://www.psychiatry.org/dsm5, accessed on 1 February 2026) and ICD (https://www.who.int/standards/classifications/classification-of-diseases, accessed on 1 February 2026)). Sensory issues and motor difficulties [1,2] are also recognized features and are included in the diagnostic criteria (DSM-5) [3]. ASD manifests early in life, with a strong sex bias (males:females = 3:1), and frequently coexists with gastrointestinal disturbances, immune dysregulation, sleep and metabolic disorders, and anxiety [4]. ASD heritability is estimated at ~80% [5], involving thousands of genetic variants, including single nucleotide polymorphisms, de novo mutations, and copy number variants affecting up to ~1200 risk genes, as reported in SFARI Gene (https://gene.sfari.org/, accessed on 13 November 2023) (a database for the autism research community). ASD is classified into syndromic forms, which are linked to specific neurological syndromes and causative mutations (e.g., Fragile X, Rett, and Angelman syndromes), and nonsyndromic forms (NS-ASD), which account for most cases. Although the etiology of NS-ASD remains uncertain, gene–environment interactions may contribute to its complexity and increasing prevalence: 1 in 33 in the USA [6] and 1 in 100 worldwide [7]. Exposure to environmental factors such as advanced parental age, maternal infections during pregnancy, and severe birth complications [8] as well as chemical pollutants [9] and intestinal pathogens [10] during the prenatal period and early life may trigger ASD. Environmental factors may cause ASD by inducing neuroinflammation, oxidative stress, epigenetic modifications or damaging DNA, ultimately impairing neurotypical brain development. These factors rarely cause autism alone but act in combination with genetic susceptibility. Despite their heterogeneity, ASD-related molecular disruptions converge on pathways affecting transcription, translation, epigenetics, synaptic function, and immune responses, all of which impact the neurobiological mechanisms underlying social behavior [11].

Social behavior, which is fundamental to animal fitness and evolutionary success, is orchestrated by complex neural networks and modulated by neuroendocrine signals. These systems are regulated by genetic and epigenetic mechanisms and shaped by environmental inputs throughout development. In both animals and humans, social cognition and interactions are mediated by key neurotransmitter systems, including dopamine, serotonin, β-endorphin, and the oxytocin–vasopressin (OXT–AVP) axis, which act on brain regions such as the prefrontal cortex, amygdala, and hypothalamus [12,13].

The OXT–AVP system plays a central role in modulating social bonding, affiliative behaviors, emotional regulation, and stress responses by interacting with serotonergic and dopaminergic pathways. Additionally, motor systems contribute to social interaction by coordinating cospeech/vocal gestures and fine-tuning adaptive behaviors [14,15,16].

At the cellular level, synaptic plasticity and neurodevelopmental pathways are important for the emergence and refinement of social cognition. These processes are under genetic control; for example, in humans, *FOXP2* and *MCPH1* are involved in language processing and brain size regulation, respectively, whereas other genes, such as *ARHGEF11*, *ZO-1*, *HCRT*, and *NCEH1*, regulate neuronal growth, synaptic remodeling, and metabolic activity [17,18,19,20].

Some social behaviors, including the complex social cognition characteristics of humans, are rooted in evolutionarily conserved molecular and neural mechanisms. For example, a dosage imbalance in the 7q11.23 locus, comprising 28 genes, differentially affects sociability across species. In humans, deletion at this locus causes Williams–Beuren syndrome (WBS) (OMIM #194050), characterized by an empathic personality open to engaging with people [21,22], whereas its duplication leads to WBS duplication syndrome (OMIM #609757), which is associated with deficits in social behavior and autism. Notably, in dogs, mobile element insertions in the corresponding genomic region, the “canine WBS locus”, reduce the expression of the sociability-related genes *GTF2I* and *GALNT17*, increasing social behavior and discriminating dogs from wolves [23,24]. These convergent effects across taxa highlight how homologous genetic regions can shape social phenotypes in evolutionarily distant species. Similarly, the human language-related genes *FOXP2* and *CNTNAP2* are functionally conserved in songbirds, where they modulate vocal learning [25,26,27]. Specifically, in parrots, renowned for vocal learning and complex sociability, genes involved in social behavior include 11 key genes linked to brain function (*AUTS2*, *BCL11A*, *EBF3, ERBB4*, *ESRRG*, *NPAS3*, *PROX1*, *SOX6*, *SP8*, *ZC3H3*, *ZNF608*) [28], four of which (*AUTS2*, *BCL11A*, *EBF3* and *SOX6*) are also implicated in ASD (SFARI (https://gene.sfari.org/, accessed on 13 November 2023)).

Deficits in social behavior highlight the role of specific neural and genetic networks. Many ASD-linked genes, such as *FOXP2*, *ARHGEF11*, *MCPH1*, and *PCLO*, regulate cognitive and social functions, whereas other genes encode for OXT neuropeptide and OXT, AVP, serotonin and dopamine receptors (OXTR, AVPR1B, 5-HT1B, DRD2, respectively) [29,30,31], all central to social interaction.

Human sociality also relies on human accelerated regions (HARs), short, highly conserved genomic sequences that have undergone rapid evolutionary changes in humans in terms of a high rate of nucleotide substitutions, leading to human–chimpanzee divergence. HARs function primarily as developmental enhancers and regulate gene expression in ways that influence traits fundamental to human evolution, including brain development, cognition, and behavior [32], influencing traits such as brain size, dexterity, and social cognition. For example, HAR1 is implicated in cerebral cortex development, whereas HAR2 affects limb dexterity. Notably, mutations in HARs are linked to neuropsychiatric disorders, including ASD [33,34,35].

Collectively, the findings outlined above suggest that genetic foundations of sociability have been preserved throughout evolution across evolutionarily distant species and that studying sociability-related genes in diverse organisms may provide insights into human social behavior [36] and, consequently, into autism. To explore this hypothesis, we analyzed the human interactome [37,38] to model the functional interconnections among the genes linked to sociability identified across taxa, from arachnids to humans.

Our results reveal an evolutionarily conserved core gene network involved in social behavior, encompassing pathways related to neurodevelopment, synaptic plasticity, and hormonal regulation, and suggest novel candidate ASD risk genes.

## 2. Materials and Methods

### 2.1. Literature Review

Articles reporting genes involved in sociability in animal species, including humans, were considered, specifically Spider [39]; Birds [28,40,41,42,43]; Cat [44,45]; Dog [46,47,48,49,50]; Dolphin [51]; Domesticated mammals [52]; Domesticated animals [53]; Dromedary [54]; Fox [55,56,57]; Horse [58]; Mouse [59]; and Homo s.s. [60,61,62,63].

### 2.2. Phylogenetics Tree

The phylogenetic tree was constructed via the Taxonomy browser tool of NCBI.

### 2.3. Human Interactome

Molecular interaction information was downloaded from STRING (v12, https://string-db.org/cgi/download, accessed on 1 October 2023) [64]. The combined score was updated, excluding “text mining” via a modified version of the script “combine_subscores.v2.py” (https://stringdb-downloads.org/download, accessed on 1 October 2023). Ensembl identifiers were mapped to Entrez Gene identifiers via the mapping available in STRING (https://string-db.org/cgi/download, accessed on 1 October 2023) and Entrez Gene (ftp://ftp.ncbi.nih.gov/gene/DATA, accessed on 19 September 2023). The highest score was considered for each gene pair. Only high-confidence (combined score ≥ 700) interactions and the top 3 (per gene) interactions with medium confidence (STRING score ≥ 400) were considered, resulting in a total of 174,962 interactions involving 17,288 genes.

### 2.4. Molecular Pathways

Molecular pathways were downloaded (20 November 2023) from Reactome [65]. Only pathways with at least 3 genes and at most 200 genes were considered for enrichment analysis, which was performed via the hypergeometric test. *p* values were corrected via the Benjamini–Hochberg procedure [66,67]. For the definition of meta-pathways (clusters of similar pathways), pathways enriched in network genes (at least 3 networks and fdr < 0.05) were used to define a network of pathways (R package igraph, version 2.1.4) [68], where an interaction between two pathways existed if the overlap coefficient between them was greater than 0.2, a value that determined the highest modularity of the pathway network (0.26) and 5 meta-pathways, that is, clusters of pathways (fastgreedy algorithm [69] in igraph).

### 2.5. Network Analysis of Genes Associated with Sociability

Network analysis was performed via the R packages “dmfind” [70] (Supplementary Methods) and igraph [68]. The initial gene weights $x_{0}$ for the network diffusion (ND) process were set to 1 for sociability genes and to 0 for all other genes. Given the symmetrically normalized adjacency matrix W=D−12AD−12, the steady state ND values

$$x_{s}=\left(1-\alpha \right){\left(I-W\right)}^{-1}x_{0}$$

were used to define the network smoothing index of gene j

$$S_{i}\left(\varepsilon \right)=\frac{x_{s}(i)}{x_{0}(i)+\varepsilon }.$$

The ND weighting parameter α was set to 0.7, as in previous works [70,71]. The parameter ε was set to 50, a value that maximized, among the top-ranking genes by decreasing $S_{i}\left(\varepsilon \right)$, the number of connected genes with high $x_{0}\left(j\right)$ (here indicating involvement in sociability), as in a previous study [71] (Supplementary Figure S1). A total of 1000 permutations were used to calculate the permutation-adjusted network smoothing index

$$\tilde{S_{i}}=-S_{i}\cdot \log_{10}\left(p_{i}\right),$$

where $p_{i}$ is the *p* value associated with $S_{i}$, and to perform network resampling. To extract the network of genes associated with sociability, the networks composed of the top r∈[50, 500] ranking genes by decreasing $\tilde{S_{i}}$ were scored on the basis of modularity, the number of genes with $p_{i}<0.01$, the *p* value associated with network resampling, the *p*-value associated with network enrichment in ASD genes, and the network with the highest score (Supplementary Figure S2).

### 2.6. Analysis of Genomic Location

The genomic locations of the genes were obtained from the R package org.Hs.eg.db (v3.14). All genes located within 50,000 bp up/downstream of a gene were linked with the gene, assuming 1 cM ≈ 106 bp. HAR genes were collected from the studies of Capra [72] and Keough [73].

## 3. Results

### 3.1. List of Genes Involved in Animal and Human Sociability and ASD

**Figure 1:**
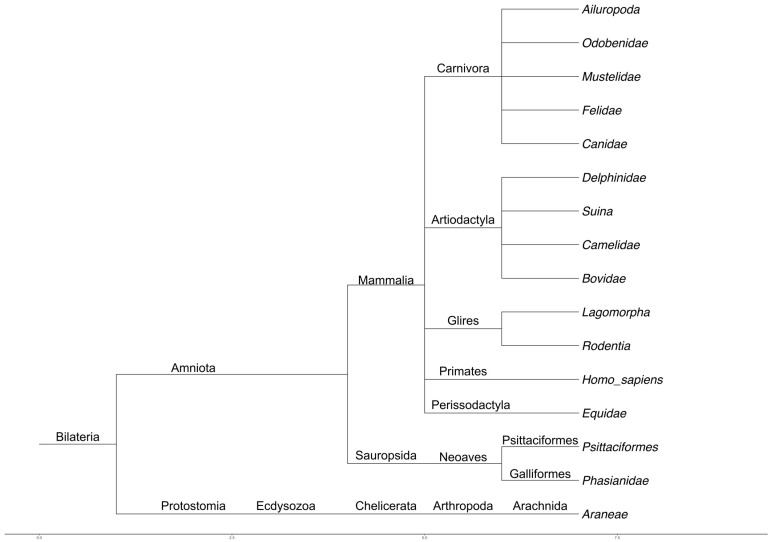
Phylogenetic tree of the animal taxa considered in the study.

**Figure 2:**
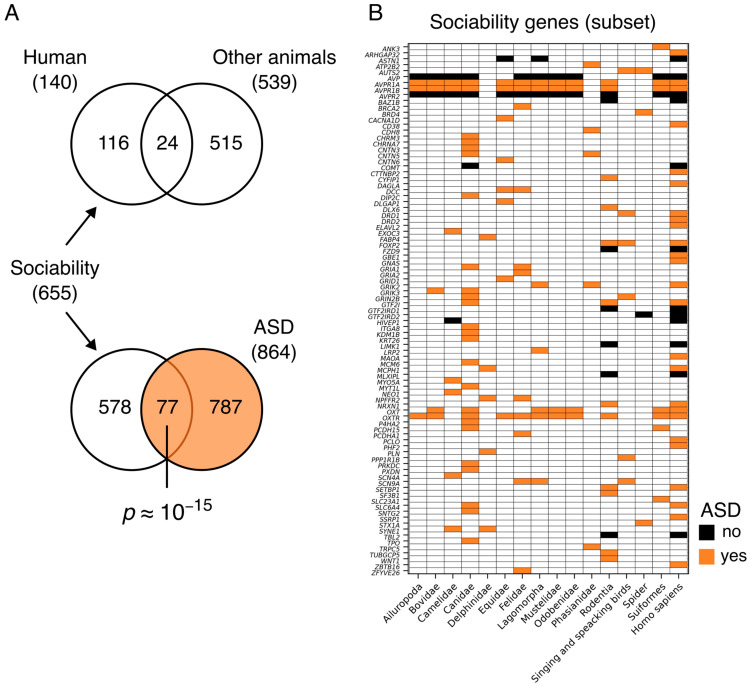
Human genes associated with sociability in humans and/or other animals. (**A**) Genes associated with sociability (in humans and other animals) (top) and with ASD (bottom); the *p* value comes from the hypergeometric test (Supplementary Table S2). (**B**) Subset of the 655 genes associated with sociability: only the 89 genes that appear in at least two out of the three categories “human”, “other animals” and “ASD”, are shown (full list in Supplementary Table S1). (**A**,**B**) Gene association with ASD is based on SFARI Gene.

By reviewing the literature concerning the genetic basis of sociality in different animal taxa, including humans, we found 27 articles (see M&M, Section 2.1, Literature review) referring to 16 evolutionarily distant genera (Figure 1) for a total of 655 sociality-related genes, of which 515 genes were specific to animal sociality, 116 to human sociality, and 24 were shared between the two groups (Figure 2A; Supplementary Table S1). Strikingly, a substantial number of genes, 77 out of 655 social genes (approximately 12%, *p* ≈ 10^−15^; Supplementary Table S2), are reported in the SFARI Gene as associated with NS-ASD (Figure 2A). More specifically, (Figure 2B), the six sociality genes owing to the OXT–AVP system (*AVP*, *AVPR-1A* and *AVPR-1B*, *AVPR2*, *OXT* and *OXTR*) are shared among almost all the mammals considered here (Ailuropoda, Bovidae, Camelidae, Canidae, Equidae, Felidae, Lagomorpha; Mustelidae, Odobenidae, Rodentia, Suiformes, and Homo s.s.); nine out of the 215 genes linked to the social behavior of Canis lupus familiaris are involved in human sociality (those six of the OXT-AVP system as well as *COMT*, *GTF2I* and *SLC6A4*), whereas 25 are reported in SFARI. Five genes (*FABP4*, *MCPH1*, *NPFFR2, PLN*, *SYNE1*), which are linked to the social behavior of Delfinidae, are reported in the SFARI, and among these genes, *MCPH1* is also linked to human sociability. Six of the 38 genes linked to the social behavior of singing and speaking birds (*AUTS2*, *DRD1*, *FOXP2*, *GRIN2B*, *PPP1R1B*, and *SCN9A*) are also ASD risk genes (SFARIs), and among these genes, *AUTS2* is also linked to the sociability of spiders, whereas *SCN9A* is linked to Felis catus and Lagomorpha. The *AUTS2*, *BRD4* and *STX1A* genes are linked both to the sociality of spiders and to ASD, whereas the *GTF2IRD2* gene is associated with spider and human social behavior.

### 3.2. Gene Networks and Pathways Associated with Human Sociability and Their Links with ASD

To reconstruct the human gene networks composed of sociability genes, we applied the gene module identification approach “dmfind” [70] (Supplementary Methods) to the human interactome derived from the database STRING [64], using 655 sociability genes (humans and human-homologous animal sociability genes) as inputs for the network diffusion process. We ranked the genes by decreasing values of the permutation-adjusted network smoothing index ($\tilde{S}$), a gene-level quantity that summarizes the network proximity of the gene to sociability genes. Based on gene ranking, we extracted, from the whole interactome, a gene network that maximizes the following variables: presence of ASD genes, network modularity, network proximity of genes with high $\tilde{S}$ values and statistical significance of $\tilde{S}$ values (Supplementary Figure S2).

**Figure 3:**
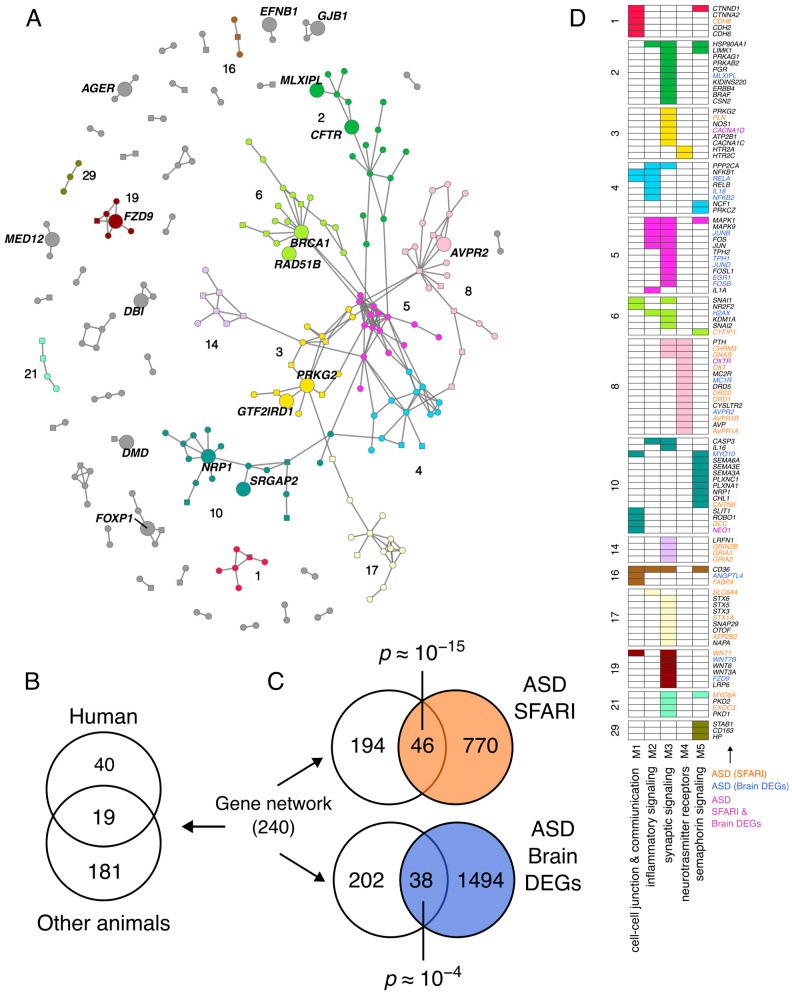
Sociability gene network in humans. (**A**) Network of genes associated with sociability. (**B**) Classification of network genes by organism where there is evidence of association with sociability. (**C**) Overlaps between network genes and genes associated with ASD or genes whose expression changes between ASD patients and neurotypical subjects (postmortem brains); the *p* values are from the hypergeometric test (Supplementary Table S2). (**D**) Network genes grouped by network community (numbers on the left) and functionally annotated by meta-pathway (M1–M5, columns).

We obtained a total of 240 sociability genes and 299 links, which were organized into a major connected component of 134 genes and 14 smaller components (from 2 to 6 genes each) (Figure 3A, Supplementary Tables S3 and S4). The network involves 59 human sociability genes, 200 human-homologous animal sociability genes, and 19 genes associated with sociability in humans and other animals (Figure 3B). It is highly enriched in NS-ASD genes, which total 46 (*p* ≈ 10^−15^, Figure 3C, Supplementary Table S2).

From a topological point of view, the network shows a modular structure (*Q* = 0.86, multilevel modularity optimization algorithm [74], with a total of 48 communities, of which 9 belong to the major connected component (Figure 3A). This modular structure suggests the involvement of various pathways.

To assess the biological functions associated with the gene network, we performed Pathway Over Representation Analysis (ORA) with Reactome [65] and, indeed, found enrichment in multiple pathways (Supplementary Table S5). To summarize the results of the pathway analysis, we clustered the significant pathways (FDR < 0.05, hypergeometric test) by their similarity (overlapping of genes) and obtained 5 meta-pathways, namely, cell–cell junctions and communication, inflammatory signaling, synaptic signaling, neurotransmitter receptors, and semaphorin signaling (Figure 3D and Figure S3, Supplementary Tables S6 and S7).

**Figure 4:**
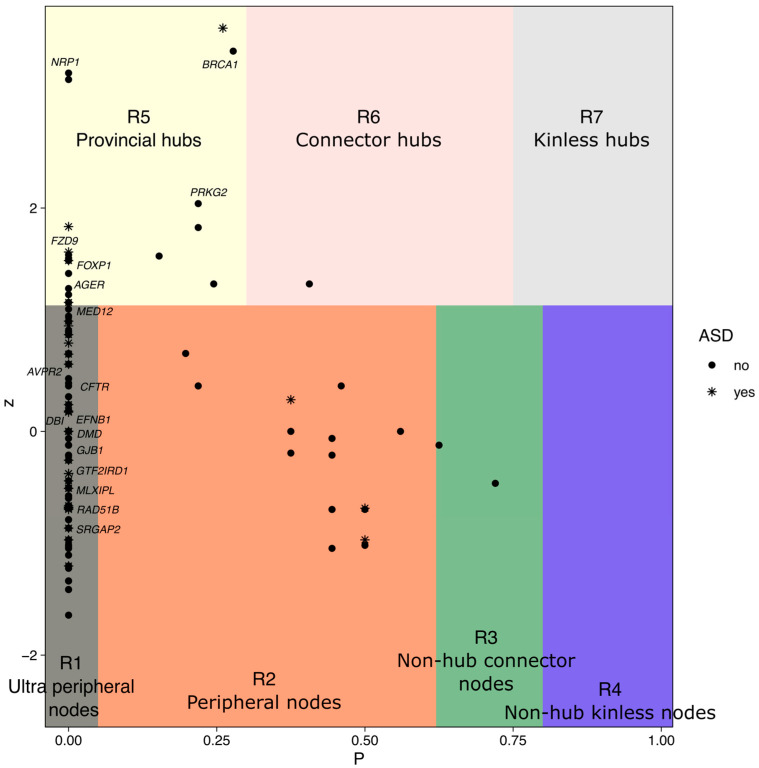
Functional cartography of the sociability gene network. Each gene is placed according to its participation coefficient (P) and within-community number of interactions (z).

To classify genes based on their topological properties within the gene network, we reconstructed their functional cartography [75]. This map allows us to classify genes into 3 types of hubs and 4 types of nonhubs (Figure 4) based on the participation coefficient, which is proportional to the number of communities a gene relates to, and the within-community number of interactions.

### 3.3. Hub Genes in the Largest Connected Component

The largest connected components contain a total of 15 genes that are classified as hubs in the functional cartography: 14 provincial hubs (PHs), which are genes with the most links within their community; and 1 connector hub, a gene with many links to most of the other communities. Here, we highlight the most interesting genes based on function, function of the gene community (Supplementary Table S3), association with ASD (both genetic and transcriptional), chromosomal localization, linkage with ASD-related genes, and HAR regulation. Interestingly, 4 provincial hubs have already been reported to be associated with ASD. The guanine nucleotide-binding protein G(S) subunit alpha isoform short (GNAS) (community 8, 13 interactions, ch20q13.32) is functionally essential for the GPCR signaling pathway, a key mechanism for cellular communication and response to external signals. The community includes pathways centered on signal transduction, hormone (OXT-AVP signaling system) and neurotransmitter (dopamine) receptor functions, and metabolic regulation. *Syntaxin 1A* (*STX1A*) (community 17, 7 interactions, WBS locus) is implicated in synaptic transmission by mediating the fusion of synaptic vesicles with the presynaptic membrane. The genes and pathways of community 17 ensure the precise delivery, modification, and localization of molecules needed for diverse cellular processes, from immune signaling to sensory function (e.g., interleukin signaling or sound processing) and neurotransmitter release. *Glutamate Ionotropic Receptor AMPA Type Subunit 2* (*GRIA2*) (community 14, 5 interactions, ch4q32.1) is a glutamate receptor that encodes a subunit of the AMPA receptor critical for fast excitatory neurotransmission in the brain that participates in synaptic plasticity, learning, and memory. *GRIA2* interacts with genes and pathways involved in the molecular and cellular mechanisms underlying synaptic plasticity, which are essential processes for learning, memory, and neuronal development. *Calcium voltage-gated channel subunit alpha 1D* (*CACNAD1*) (community 3, 6 interactions, ch3p21.1) encodes the alpha 1D subunit of the channel that regulates calcium ion entry into excitable cells and plays a key role in numerous calcium-dependent functions, such as muscle contraction, the release of hormones or neurotransmitters, and the regulation of gene expression. It interacts with pathways interconnected through their roles in signal transduction, homeostasis, metabolism, and sensory function, forming a network that supports core physiological processes across various organ systems.

Other PHs are not directly related to NS-ASD, although some other genes included in their community are associated with this condition.

*Catenin delta 1* (*CTNND1*) (community 1, 4 interactions, ch11q12.1) encodes a protein that functions in adhesion between cells and signal transduction. The pathways of community 1 are centered mainly around cell–cell adhesion, junction organization, and the regulation of cadherin function, which are essential for structural integrity, signaling, and communication between cells, especially in the context of tissue formation, maintenance, and repair. *Breast cancer gene 1* (*BRCA1*) (community 6, 12 interactions, DEG in the brain of autistic individuals, ch17q21.31): involved in maintaining genomic stability and ensuring cellular integrity. It interacts with genes related to pathways related to cell cycle regulation, epigenetic regulation, and cellular homeostasis. *AP-1 transcription factor subunit* (*JUN*) (community 5, 12 interactions, ch1p32.1): a transcription factor that plays a key role in cell proliferation, differentiation, apoptosis, and stress responses. It is a provincial hub of a broad network controlling senescence and stress adaptation, immune and inflammatory responses, and cellular transcriptional control. *Protein kinase CGMP-dependent 2* (*PRKG2*), community 3, 8 interactions, HAR ch14q32.31), act downstream of NMDAR to activate the plasma membrane accumulation of GRIA1/GLUR1 in synapses and increase synaptic plasticity. The pathways included in the community play roles in signal transduction, homeostasis, metabolism, and sensory function. *Neuropilin 1* (*NRP1*) (community 10, 8 interactions, HAR ch10p11.22) encodes one of two neuropilins, a coreceptor for multiple signaling pathways involved in axon guidance, angiogenesis, immune regulation, and tumor progression; the pathways represented in the community are related to neuronal connectivity and intercellular communication, particularly in the context of neuronal development and signaling.

### 3.4. Relevant Genes in Smaller Network Components

For the 14 smaller connected components, the relevant genes of the communities, since they are involved in homeostatic processes, neural circuits, metabolism and the immune system, are as follows:

*Frizzled class receptor 9* (*FZD9*) (community 19, 5 interactions, DEGs in the ASD brain, owing to the WBS locus). These pathways are involved in receptor-mediated signaling, trafficking of signaling molecules, and regulation of developmental and homeostatic processes.

*Mediator complex subunit 12* (*MED12*) (community 7, R5, 2 interactions, DEG in the ASD brain, chXq13.1). The genes of this community play a role in regulating lipid utilization, storage, and energy metabolism at both the transcriptional and systemic levels

*Forkhead Box P1* (*FOXP1*) (community 26, R5, 3 interactions, under HAR control, ch3p13). It is a transcription factor that participates in nervous system development. *FOXP1* variants are associated with global and speech delays and intellectual disability

*Diazepam binding inhibitor* (GABA Receptor Modulator, Acyl-Coenzyme A Binding Protein) (*DBI*) (community 23, R1, 2 interactions, DEG in ASD brain, ch2q14.2). The pathways of plasma lipoprotein assembly, remodeling, and clearance regulate the processes by which lipoproteins are formed, modified, and eventually removed from the bloodstream, making them critical for lipid metabolism and energy homeostasis.

*Advanced glycosylation end-product specific receptor* (*AGER*) (community 25, 2 interactions, DEG in the ASD brain, ch6p21.32). It is an immunoglobulin superfamily of cell surface receptors involved in development, homeostasis and inflammation and is implicated in diabetes and Alzheimer’s disease.

### 3.5. Differentially Expressed Genes in the Postmortem Brains of Autistic Individuals Compared with Those of Neurotypical Subjects

To assess whether other network genes could be linked with ASD beyond genetics, we considered the 1570 differentially expressed genes (DEGs) in the postmortem brains of ASD patients in comparison with the neurotypical subjects reported by Morton JT and collaborators [76] (Supplementary Table S9). Interestingly, we found that a significant number of the top network genes (38, *p* ≈ 10^−4^) were DEGs, rising for 78 (33%) of the total number of network genes linked to ASD to 78 (SFARI + DEGs, (Figure 3C, Supplementary Table S2). Among the sociability network genes that are also DEGs in ASD brains, 6 genes (*CACNA1D*, *CD38*, *MAOA*, *NEO1*, *OXTR*, and *PPP1R1B*) are both DEGs and NS-ASD (SFARI), whereas 5 inferred genes (*BRCA1*, *FZD9*, *MED12*, *DBI* and *AGER*) are peripheral hubs of the abovementioned communities #6, 19, 7, 23, and 25, respectively, as well as two (*JUNB* and *JUND*) paralogs of *JUN*, the PH of community 5. In addition to *MED12*, three other inferred genes map on the X chromosome with possible implications for the sex bias of ASD: ephrin B1 (*EFNB1*), which may act in cell adhesion and neurodevelopment or neuro-maintenance; *vasopressin receptor type 2* (*AVPR2*), which is involved in the OXT-AVP system; and *Dystrophin* (*DMD*), whose variants cause Duchenne and Becker muscular dystrophy, which often accompany ASD [77].

### 3.6. Chromosomal Bands That Are Enriched in Network Genes

From a physical standpoint, we assessed whether there are chromosomal bands that are enriched in genes that compose our network. We found 12 chromosomal bands enriched in network genes, nine of which were statistically significant (FDR < 0.25) (Supplementary Table S10).

Specifically, ch7q11.23 corresponds to the WBS locus and includes *GTF2I* and the PH *STX1A*, both in SFARI, and the DEGs *GTF2IRD1*, *MLXIPL* and *FZD9*, which is also a PH; ch7q21.11 includes the top network inferred genes, namely, the *CD36 Molecule* (CD36 Blood Group) (*CD36*), a cell adhesion molecule, and *semaphorin-3A*, *-D* and *E* (*SEMA3-A*, *-D* and *-E*), which are implicated in neuronal development. Increased expression of *SEMA-3A* is associated with schizophrenia and the progression of Alzheimer’s disease. Ch2p25.3 encompasses three top network genes: the inferred *SH3* and *SYLF domain containing 1* (*SH3YL1*) and the ASD-related *syntrophin gamma 2* (*SNTG2*) and *thyroid peroxidase* (*TPO*). Microduplication of ch2p25.3 is associated with speech delay, behavioral disorders and ASD [78]. The Ch14q24.1 band includes the inferred genes *retinol dehydrogenase 11 and 12* (*RDH-11 and -12*), which are involved in retinoid metabolic processes and are associated with retinal diseases and the DNA repair protein RAD51 homolog 2 (RAD51B), ChXq13.1 may be involved in the sex bias observed in ASD and includes the following genes: *EFNB1*, *Gap Junction Protein*, *Beta 1* (*GJB1*), encoding a gap junction protein involved in gap junction trafficking and vesicle-mediated transport pathways and whose mutations cause peripheral neuropathy; *MED12*, the PH of community 7; and one of the top degree genes among the DEGs in the ASD brain [76].

### 3.7. Network Genes Linked with ASD Genes

To assess whether the network genes are linked with ASD genes, we considered the genomic neighbors of every network gene within 0.5 cM (before and after) and evaluated the presence of ASD genes (Supplementary Table S11). It is likely that the *MED12* (chXq13.1) and *GJB1* (chXq13.1) genes, which immediately precede and follow the ASD-related *NLGN3X* gene, are inherited along with it. *NLGN3X* encodes a protein that acts as a cell adhesion molecule and plays a critical role in synaptic signaling and neuronal communication.

In contrast, the *SLIT-ROBO Rho GTPase Activating Protein 2* (*SRGAP2*) gene, which is involved in the regulation of neuronal development, is located between two ASD-associated genes: *arginine vasopressin receptor* (*AVPR1B*) and *Ras Association Domain Family Member 5* (*RASSF5*), a tumor suppressor gene involved in apoptosis and cell cycle regulation. Interestingly, *SRGAP2* is known for its role in human brain evolution: it underwent duplications in the human lineage after it split from its common ancestor with chimpanzees. These duplications resulted in the creation of additional copies, specifically *SRGAP2C*, which is a partial duplication that can interact with the original *SRGAP2* gene product. This interaction has been suggested to slow down neuronal development, potentially leading to a longer period of cortical development and an increase in cognitive ability [79]. The *CF transmembrane conductance regulator* (*CFTR*) gene maps at ch7q31.2 immediately before the *CTTNBP2* gene. *CTTNBP2* regulates dendritic spine formation, distribution and maintenance, and its deficiency is associated with ASD [80]. *AVPR2* (chXq28) is tightly linked to and should be inherited together with *MECP2*, from which it is approximately 105 bp in length. Mutations in *MECP2* cause Rett syndrome, an X-linked rare neurodevelopmental disorder occurring in females only and that, although no longer classified as ASD, shares many features and molecular convergence with it [81].

### 3.8. Network Genes Regulated by HARs

Finally, 24 network genes are part of the HARs (Supplementary Table S3), of which 9 are reported in SFARI Gene. Among the other 15 genes, we identified PH *NRP1* and *PRKG2*, the DEG *DMD* on chX43; *catenin alpha 2* (*CTNNA2*) and nuclear receptor subfamily 3 group C member 2 (*NR3C2*), associated with syndromic autism; *RAD51B* at the enriched locus Ch14q24.1; *Erb-B2 receptor tyrosine kinase 4* (*ERBB4*), encoding a Tyr protein kinase; *FRAS1-related extracellular matrix 1* (*FREM1*), which may regulate Toll-like receptor/interleukin 1 receptor signal transduction; the transcription factor *paired box 3* (*PAX3*); *roundabout guidance receptor 1* (*ROBO1*), encoding a protein that functions in axon guidance; and *tyrosinase* (*TYR*), whose product is involved in the conversion of tyrosine to melanin.

## 4. Discussion

Understanding the genetic basis of sociality is crucial for gaining insights into the etiopathogenesis of ASD, for which a strong hereditary component has been demonstrated. Social behavior is a complex and evolutionarily conserved trait that is influenced by the interaction of multiple genetic and environmental factors. Alterations in genes and pathways that regulate social functioning contribute to the emergence of ASD, and molecular variants may explain the heterogeneity in ASD symptoms and severity. Therefore, identifying evolutionarily conserved genes that play a role in regulating social behavior not only enhances our knowledge of typical neurodevelopmental processes but also provides valuable insights into the neurotypical processes of social interaction and the variants related to ASD.

Animal models serve as valuable tools for understanding biological processes, such as molecular pathways and cell–cell interactions, in both healthy and disease conditions and developing potential treatments such that selecting adequate models for studying human phenomena is indeed a complex and crucial task in scientific research. Social behaviors result from complex interactions of a multitude of biological pathways that are genetically and environmentally driven, so the choice of animal models to recapitulate human sociability and related disorders, such as ASD, is even more challenging. Several aspects must be considered when choosing the right animal model for a particular human event, such as genetic and anatomical similarity, cross-species translatability, availability, and practicality. To date, no animal model is a perfect representation of human biology and behavior, and researchers often use a combination of models to address different aspects of a condition. Indeed, the translational value of rodent models, which are currently the pivot in analyzing molecular mechanisms of ASD and testing novel compounds, is questioned, whereas nonhuman primate models, especially those with great potential, may have greater translational power but are limited by practical constraints such as the poor availability of subjects and ethical concerns [82].

In this context, the wide comparative approach has become increasingly important in research on human behavior since shared genetic elements, which influence social relationships across multiple animal species, aligns with the broader field of evolutionary biology and genetics. Indeed, evolution might have preserved certain genetic pathways involved in sociality that confer adaptive advantages in dealing with social environments. The comparative approach allows the exploration of the neural, biological, and genetic basis of behavior and cognition and the identification of shared mechanisms of social interactions, communication, cooperation, and adaptations that are useful for understanding how certain social abilities have developed over time or can be impaired in specific human conditions [83,84].

A review of the literature revealed that some genes linked to human sociability [23] occurred in more than one animal species, even those that are evolutionarily distant, and that a subgroup of them are involved in ASD. In this context, we tested the hypotheses that some genetic pillars of sociality belong to a network that is evolutionarily stable enough across animal species and that they could be useful not only for understanding the genetic basis of human behavior but also for inferring novel risk genes for autism. For this purpose, a network-based approach was applied to the list of 659 genes involved in sociability across evolutionarily distant animal species, including humans, which first identified a network of ~17,000 genes and, within this network, a top network enriched with 240 more significantly interconnected genes. This top network is made up of 23 communities (also called modules). The modular structure highlights the cooperative and organized nature of genetic interactions, which often leads to a better understanding of biological functions and disease mechanisms. In a pathological context, identifying communities can help identify key regulatory genes or pathways that could be targeted for therapeutic interventions. Nine of these communities interact with each other in a major connected component, whereas 14 constitute smaller single connected components.

The significant pathways identified in the gene communities highlight key biological processes, particularly those related to the nervous system, immune response, metabolism, and cell communication. With respect to neuronal function, we identified pathways related to neurotransmitter receptors, postsynaptic signaling, and synaptic transmission (community #14), whereas axon guidance and nervous system development are central to community #10. Sensory perception, particularly auditory processing, is linked to #3 and #17. Cell communication and adhesion refer to #1, which is associated with pathways of cell–cell communication, adherens junctions, and junction organization, ensuring tissue integrity and function. The immune system and inflammation involve communities #4, #5, #11, #16, and #25, which include pathways of both innate and adaptive immune responses, pathogen recognition, and inflammation, which are notably linked to neuroinflammation in autism spectrum disorder (ASD). Metabolism and hormone signaling refer to lipid metabolism represented in #7, #15, #16, and #23, whereas hormone signaling, including secretin family receptors, appears in #19. Cellular stress and transport include #4, #5, and #15, which are involved in the response to stress and stimuli. Membrane trafficking and vesicle-mediated transport are significant in #21, #29, and #32. Key signaling pathways are represented by WNT signaling, which is crucial for cell regulation and development and is significant in #19. GPCR-related signaling, including neuropeptides associated with social behavior (*OXC*, *OXCR*, *AVP*, *AVPR*), appears in #8 and #19, whereas neurotransmitter signaling is noted in #30. These findings emphasize the interplay between neuronal function, immune responses, metabolism, and cell signaling in the studied communities and in relation to social behavior.

For sociality genes shared among different animal genera, some genes owing to the OXT–AVP system (*AVP*, *AVPR-1A* and *AVPR-1B*, *AVPR2*, *OXT* and *OXTR*) are common to almost all mammals considered in the study. The *AUTS2*, *DRD1*, *FOXP2*, *GRIN2B*, *PPP1R1B*, and *SCN9A* genes are involved in the social behavior of singing and speaking birds and autism, whereas the *AUTS2*, *BRD4* and *STX1A* ASD genes are linked to both spider sociability and ASD.

From a physical standpoint, 37 out of 194 inferred ASD risk genes are clustered with statistical significance within nine chromosomal bands, each encompassing three to 10 of these genes (Supplementary Table S10). Those with the strongest statistical significance are mapped at 7q11.23, corresponding to the WBS locus; 7q21.11, whose deletion has been associated with generalized seizure and autistic traits [85]; and Xq13.1, with possible implications for the sex bias observed in ASD. Gene clustering indicates functional cooperation, shared evolutionary history, and coordinated regulation contributing to the robustness and adaptability of complex biological traits.

Moreover, 45 of the inferred genes map within less than 0.5 cM of the ASD-associated genes, sometimes in clusters of up to four, revealing a strong genetic linkage to these autism-related genes, with which it is highly probable that they will be inherited together (Supplementary Table S11).

Concerning HARs, we found that HARs epigenetically control 24 network genes, 15 of which are inferred (Supplementary Table S3) to act as human-specific neurodevelopmental enhancers.

Taken together, these findings revealed stronger inferred risk genes for autism: *MED12*, a regulator of gene transcription with a key role in neural development; since it is a PH (community #7), it is one of the top degree genes among the DEGs of the brain of ASD individuals, belongs to a cluster of genes located with statistical significance on chXq13.1, is linked with the ASD-related gene *NLGN3X* and is also associated with X-linked disorders, including intellectual disability; *FZD9*, a receptor for WNT signaling proteins, is a PH (#9), a DEG of the ASD brain, a member of the cluster genes that maps in the WBS locus, and is involved in WBS and Alzheimer’s disease; and *DMD*, since it is a DEG in the ASD brain, maps on chX and is under HAR epigenetic control. These genes are followed by PHs *PRKG2*, *NRP1* and *FOXP1* (linked to syndromic ASD), which are related to HARs; PHs *BRCA1*, *DBI* and *AGER*, which are all DEGs in the brains of ASD individuals; *GTF2IRD1* and *MLXIPL*, which are both DEGs and located in the WBS locus; *EFNB1*, which is a DEG owing to the cluster on chXq13.1; *AVPR2*, a member of the OXT-AVP system, and a DEG located on chX; *RAD51B*, a member of the cluster mapping on Ch14q24.1 and related to a HAR; and *GJB1*, a member of the cluster on chXq13.1 and in linkage with the ASD-related gene *NLGN3X*.

Then again, among these stronger candidate risk genes, *FZD9*, *GTF2IRD1* and *MLXIPL* are associated with both human and animal sociability

In support of the predictive reliability of the gene network approach, we began our analysis via the SFARI gene list published in November 2023 (SFARI gene Q3 2023). Since then, four of the inferred genes (*CTNND1*, *TRPC4*, *HTR2C* and *PKD1*) have been added to the SFARI database (SFARI gene Q1 2025, nonsyndromic). Similarly, 12 genes (*CNTN3*, *CNTN5*, *CNTN6*, *DLGAP1*, *ANK3*, *NLGN2*, *GRIK3*, *CNTNAP3*, *CACNB1*, *CACNA2D1*, *RBBP5*, and *PPP2R1B*) of the 93 autism risk genes inferred by gene network analysis and published in 2017 are now listed in the non-syndromic SFARI Gene Q1 2025 [86], 3 of which are also predicted by this analysis (*CNTN6*, *DLGAP1* and *GRIK3*).

The inferred genes require in-depth investigation to determine whether autistic individuals carry genomic variants, show altered expression levels, or exhibit epigenetic modifications that affect these genes. A further multilevel validation integrating in silico, in vitro, and in vivo analyses to elucidate the direct, functional, and causal link between these genes and ASD is also needed. From a computational perspective, pathogenicity prediction tools would be useful to assess whether variants in these genes are functionally relevant; in vitro studies would evaluate the effects of manipulating the candidate genes in cell lines or brain organoids; in vivo investigations, such as the creation of knockout or knock-in animal models, would allow for functional and expression studies to confirm whether each gene functions in a tissue-specific or physiologically significant manner within a living system.

The findings of this study have several limitations. One of these is the exclusion of primates. Since the aim of this work was to discover the genomic pillar of sociability, only animal taxa that are evolutionarily distant from humans have been considered. Nevertheless, studies on the genetics of social behavior are limited to some mammals (especially pets and farm animals), a few of which are birds and spiders. Our study is based on a gene-centric molecular network. The gene–gene interactions available in the literature are aspecific, and as such, they are a model of the interactions that potentially take place in human cells. Moreover, the collection of molecular interactions is known to be affected by various biases. We used a state-of-the-art source of interactions, which we further filtered to ensure an appropriate trade-off between confidence, coverage of genes and the presence of biases, following the recommendations of previous studies. Lastly, functional annotation was limited by what was available in an earlier version of Reactome. As the curation efforts by the international scientific community to uncover gene-pathway associations go on, an updated re-analysis of the genes identified by our network-based approach could reveal some additional pathways compared to those reported in our study.

## 5. Conclusions

The results of this study further suggest that multiple animal species can serve as a more complete model to define the genomic pillars of sociability. Moreover, the integration of comparative sociogenetics data by cutting-edge bioinformatic tools allows the prediction of possible novel risk genes implicated in autism susceptibility, underlying some neurobiological mechanisms of this condition.

All the results obtained require further investigations, and the inferred genes, if validated, represent novel biomarkers of ASD.

This approach can be applied to other fields in which evolutionary preservation of fundamental genetic pathways is demonstrated.

## Data Availability

The original contributions presented in this study are included in the article/Supplementary Materials. Further inquiries can be directed to the corresponding author.

## Supplementary Materials

The following supporting information can be downloaded at https://www.mdpi.com/article/10.3390/genes17040368/s1: Supplementary Methods. Identification of the sociability gene network. Supplementary Figure S1. Optimization of parameter epsilon. Values of Omega (vertical axis) at different values of epsilon throughout the gene ranking by decreasing network smoothing index (horizontal axis). AUC is equal to O(omega). Supplementary Figure S2. Selection of the sociability gene network. Network scores (normalized to the corresponding max or min) throughout the gene ranking by decreasing permutation-adjusted NSI (horizontal axis). PRODUCT corresponds to the normnalized module score. Supplementary Figure S3. Sociability genes by pathway. Numbers on the left indicate network communities; M1-M5 indicate the meta-pathways. Supplementary Table S1. Genes associated with sociability and ASD. Supplementary Table S2. Overlap among sociability and ASD. N: universe; wb: white balls (set 1); bd: balsl drawn (set 2); wbd: white balls drawn (set 1 & set 2); exp: expected wbd; er: enrichment ratio; p_val: hypergeometric p. Supplementary Table S3. Sociability gene network-genes. Sp: permutation-adjusted network smoothing index; S: network smoothing index; p: empirical p-vlaue associated with S; subnetId: id of the connected component; subnetSize: size of the connected component; comm_id: community id; R: functional cartography label; R description: description of R; P: participation coefficient; wmd_score: within-module degree (z); animal: evidence of association with sociability in animal (human excluded); human: evidence of association with sociability in human; sfari: association with ASD; degree: number of interactions with other network genes; interactors: direct interactors of the gene in the network; HAR_Keough2024: human accelerated regions according to Keough 2024 [73]; HAR_Capra2013: human accelerated regions from Capra 2013 [72]; brain: differential expression in ASD brains from Morton 2023 [76]. Supplementary Table S4. Sociability gene network-interactions. Supplementary Table S5. Reactome pathways that are enriched in sociability genes. N: all genes of the interactome; wb: white balls (network genes); bd: balls drawn (pathway genes); wbd: white balls drawn (genes in the network and in the pathway); exp: expected value of wbd; er: enrichment ratio; p_val: hypergeometric p value; p_adj: adjusted p-value (fdr); description: pathway; genes: genes in the network and in the pathway; meta_pathway: meta-pathway ID. Supplementary Table S6. Sociability network genes, pathways and meta-pathways. comm_id: community id; meta_pathway: meta-pathway ID. Supplementary Table S7. Definition of meta-pathways. Id: meta-pathway ID; n_genes: number of genes in the meta-pathway; genes: genes in the meta-pathway; n_pathways: number of pathways; pathways: list of pathywas; meta-pathway: meta-pathway. Supplementary Table S8. Reactome pathways that are enriched in sociability genes, by community. N: network genes; wb: white balls (community genes); bd: balls drawn (pathway genes); wbd: white balls drawn (genes in the community and in the pathway); exp: expected value of wbd; er: enrichment ratio; p_val: hypergeometric p value; p_adj: adjusted p-value (fdr); description: pathway; genes: genes in the community and in the pathway. Supplementary Table S9. Genes that are expressed in ASD brain. Supplementary Table S10. Chromosomal bands that are enriched in sociability network genes. N: all citogenetic locations of genes in the interactome; wb: white balls (network genes); bd: balls drawn (genes in the cytogenetic location); wbd: white balls drawn (genes in the network and in the cytogenetic location); exp: expected value of wbd; er: enrichment ratio; p_val: hypergeometric p value; p_adj: adjusted p-value (fdr); description: cytogenetic location; genes: genes in the network and in the cytogenetic location. Supplementary Table S11. Sociability Network Genes in linkage with ASD genes. # genes in chr: number of genes in the chromosome; # genes < 0.5cM: number of genes within 0.5cM from the considered gene; # SFARI genes in chr: number of ASD genes in the chromosome; # SFARI genes in chr & < 0.5cM: number of genes within 0.5cM from the considered gene that are ASD genes; expected: expected number of genes within 0.5cM from the considered gene that are ASD genes; enrichment ratio; p_val: hypergeometric p value; p_adj: adjusted p-value (fdr); SFARI genes in chr & < 0.5cM: genes within 0.5cM from the considered gene that are ASD genes.

## Abbreviations

The following abbreviations are used in this manuscript:

ASD	Autism Spectrum Disorders
Ch	Chromosome
DEG	Differentially Expressed Genes
HAR	Human Accelerated Regions
NS-ASD	Non-Syndromic-Autism Spectrum Disorders
SFARI	Simons Foundation Autism Research Initiative
WBS	Williams-Beuren Syndrome
WHO	World Health Organization
