# Supplementary material for: Inference of Autism Risk Genes Through Comparative Sociogenomics and Molecular Network Analysis
Source: Genes (Basel). 2026 Mar 25;17(4):368. doi: 10.3390/genes17040368 (PMC13115841; doi:10.3390/genes17040368)
Supplement: Supplementary file 1 [file genes-17-00368-s001.zip › genes-4122804-Supplementary Material REVISED/Supplementary_Material.pdf]

SUPPLEMENTARY MATERIAL

Inference of Autism Risk Genes through Comparative Sociogenomics and Molecular Network Analysis

Alice Chiodi†, Ettore Mosca†, Francesca Anna Cupaioli, Alessandra Mezzelani*

National Research Council-Institute for Biomedical Technologies (CNR-ITB), Via Fratelli Cervi, 93, 20054 Segrate, MI, Italy

* Correspondence: alessandra.mezzelani@itb.cnr.it

† These authors contributed equally to this work.

TABLE OF CONTENTS

SUPPLEMENTARY METHODS **3**

IDENTIFICATION OF THE SOCIABILITY GENE NETWORK 3

Gene network scoring *3*

SUPPLEMENTARY FIGURES **5**

SUPPLEMENTARY FIGURE S1. OPTIMIZATION OF PARAMETER $\boldsymbol{\varepsilon}$. 5

SUPPLEMENTARY FIGURE S2. SELECTION OF THE SOCIABILITY GENE NETWORK. 6

SUPPLEMENTARY FIGURE S3. SOCIABILITY GENES BY PATHWAY. 7

REFERENCE **8**

SUPPLEMENTARY METHODS

Identification of the sociability gene network

The parameter ϵ was set to maximize, among the top ranking genes by decreasing $S_i(\varepsilon)$, the number of connected genes with high initial weight $\mathbf{x}_0(i)$ (indicating sociability) as previously done [1]. For a given ϵ value, define:

- the sequence of the "top ranking" genes $(g_n)_{n\in I_\varepsilon}$ corresponding to the decreasing sequence $(S_n)_{n\in I_\varepsilon}$, where $I_\varepsilon = \{1, 2, \dots, r\}$;
- the adjacency matrix $\mathbf{A}_I = (a_{ij})_{i,j\in I_\varepsilon}$;
- the non-decreasing objective function

$$\Omega(I_\varepsilon, \mathbf{x}_0) = \sum_{i=2}^{r}\sum_{j=1}^{r-1} a_{ij}\, \mathbf{x}_0(i)\, \mathbf{x}_0(j)\,;$$

- the sum of Ω values for all the subsequences $J_\varepsilon \subseteq I_\varepsilon$ composed of the top ranking $m \leq r$ genes

$$O\big(\Omega(J_\varepsilon, \mathbf{x}_0)\big) = \sum_{J_\varepsilon\subseteq I_\varepsilon} \Omega(J_\varepsilon, \mathbf{x}_0)\,.$$

We selected the value of ε corresponding to a gene ranking that maximized the presence of connected genes with high initial weight $\mathbf{x}_0(i)$

$$\underset{\varepsilon}{\operatorname{argmax}}\Big(O\big(\Omega(J_\varepsilon, \mathbf{x}_0)\big)\Big).$$

This procedure was applied to $\varepsilon \in \{0.1, 0.5, 1, 10, 50, 100, 1000, 5000\}$ on the top $r = 250$ genes. The optimal values of ϵ was 50 (Figure S1).

Gene network scoring

The network analysis produced a gene ranking by the permutation-adjusted network smoothing index $\widetilde{S_i} = -S_i \cdot \log_{10}(p_i)$, where each rank r corresponds to a gene network $G_r =$

(V_r, E_r) composed of the top r genes by $\widetilde{S_i}$. To select a gene network associated with sociability, we scored each network G_r combining topological and biological criteria:

$$y'_r = \frac{y_r}{y_{\max}} = \frac{Q_r}{\max_r(Q_r)} \cdot \frac{f_r}{\max_r(f_r)} \cdot \frac{\log_{10}(p_r^{\mathrm{NR}})}{\min_r(\log_{10}(p_r^{\mathrm{NR}}))} \cdot \frac{\log_{10}(p_r^{\mathrm{ASD}})}{\min_r(\log_{10}(p_r^{\mathrm{ASD}}))}$$

where:

- $Q_r = \frac{1}{2\,|E_r|} \sum_{ij} \left(\mathbf{A}(i,j) - \frac{d_i d_j}{2|E_r|} \right) \delta(c_i, c_j)$ is the modularity, d is the degree and c is the community;
- $f_r = \frac{|\{p_i < 0.01\}|}{|V_r|}$ is the fraction of genes with significant S_i;
- p_r^{NR} is the p-value associated with network resampling;
- p_r^{ASD} is the p-value associated with network enrichment in ASD genes.

This procedure was applied to the top $r \in \{1, 2, \dots, 500\}$ genes and the highest y'_r corresponded to $r = 320$ (Figure S2).

SUPPLEMENTARY FIGURES

Supplementary Figure S1. Optimization of parameter ε.

Values of Ω (vertical axis) at different ε values throughout the gene ranking by decreasing S (horizontal axis). AUC is equal to $O(\Omega)$.

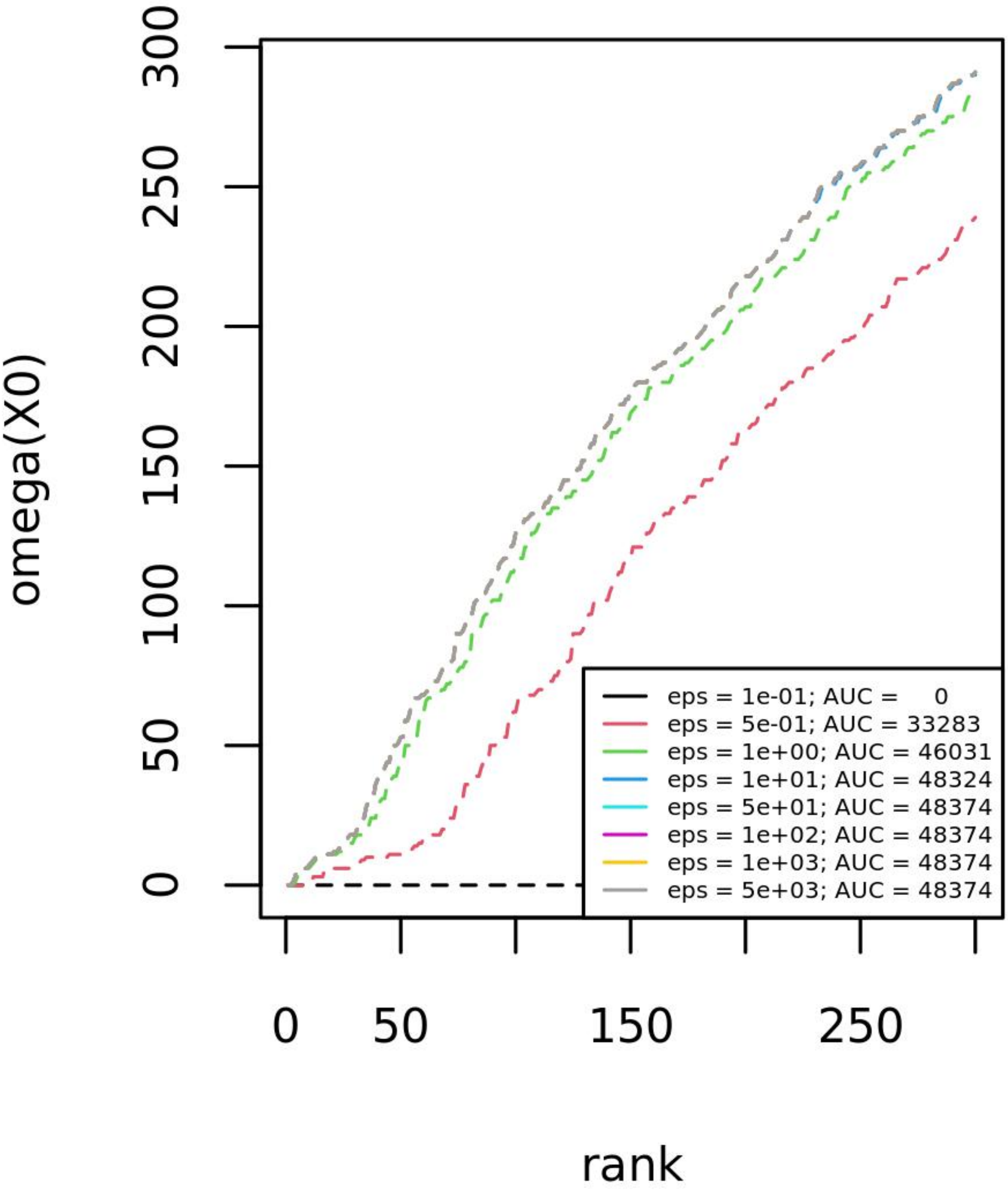

Supplementary Figure S2. Selection of the sociability gene network.

Network scores (normalized to the corresponding max or min) throughout the gene ranking by decreasing $\widetilde{S}_{\iota}$ (horizontal axis). PRODUCT corresponds to y_r'.

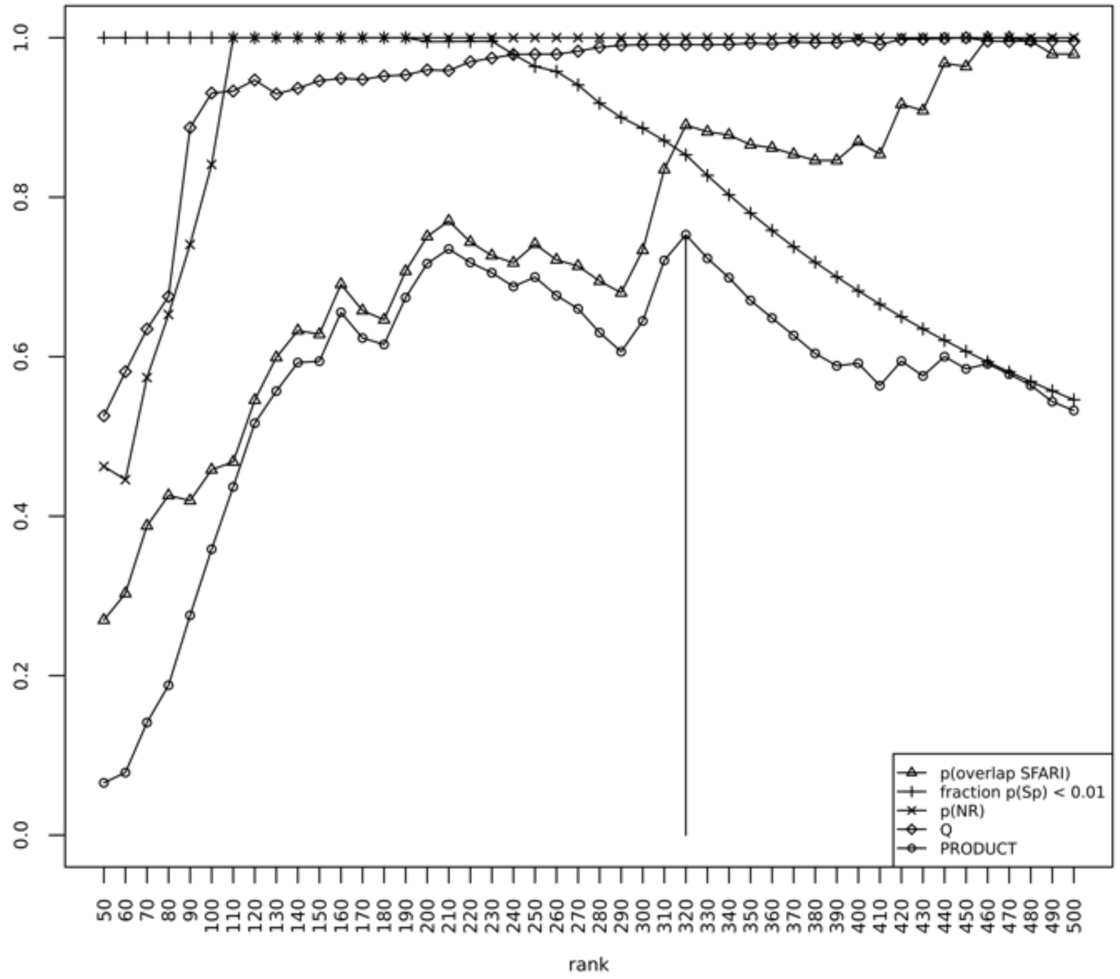

Supplementary Figure S3. Sociability genes by pathway.

Numbers on the left indicate network communities; M1–M5 indicate the meta-pathways.

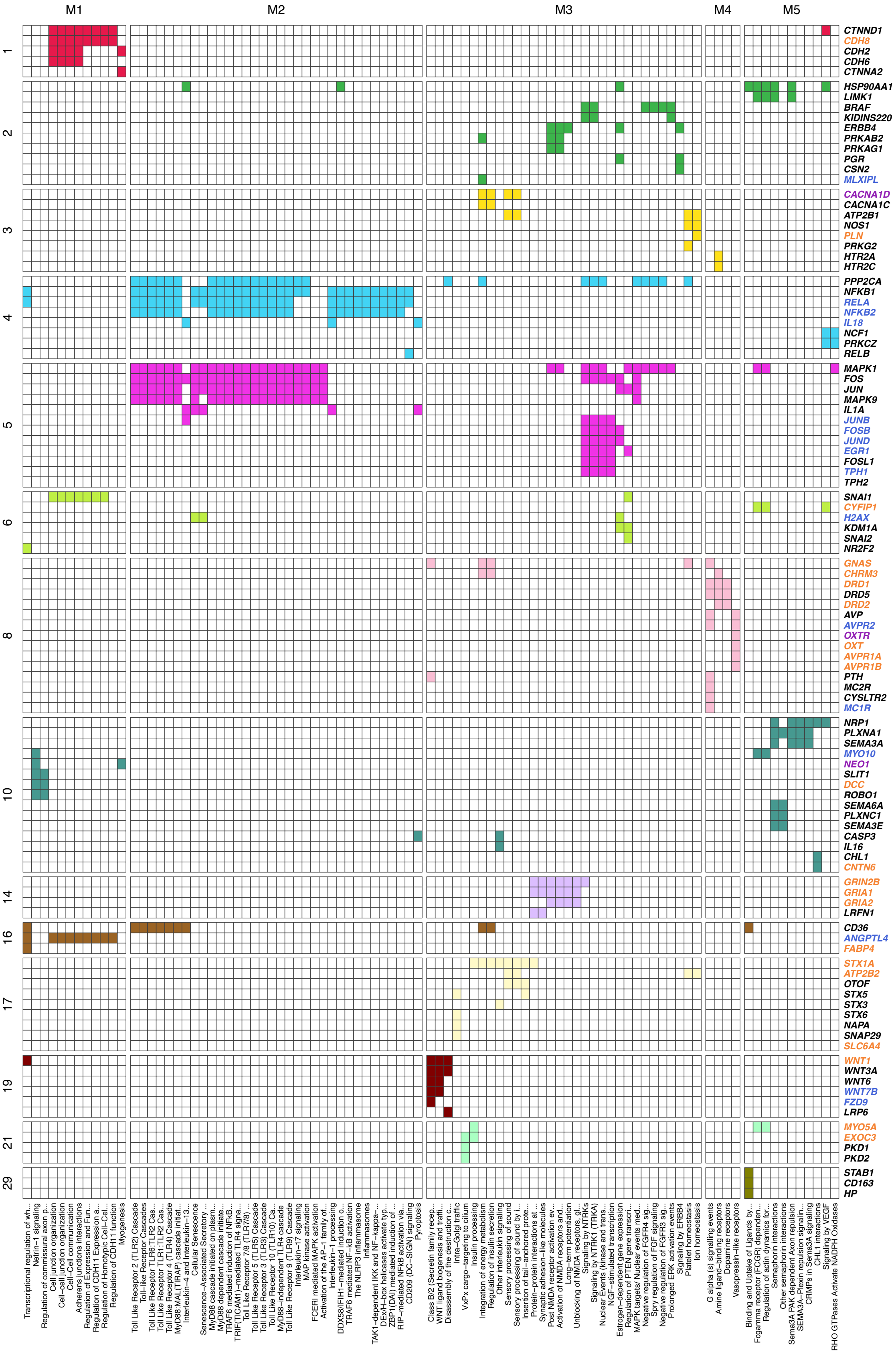

REFERENCE

1. Mascia, E.; Nale, V.; Ferrè, L.; Sorosina, M.; Clarelli, F.; Chiodi, A.; Santoro, S.; Giordano, A.; Misra, K.; Cannizzaro, M.; et al. Genetic Contribution to Medium-Term Disease Activity in Multiple Sclerosis. *Mol. Neurobiol.* **2025**, *62*, 322–334, doi:10.1007/s12035-024-04264-8.
